# Polycystic Diseases in Visceral Organs

**DOI:** 10.1155/2011/609370

**Published:** 2011-12-26

**Authors:** Shakila Abdul-Majeed, Surya M. Nauli

**Affiliations:** Department of Pharmacology, The University of Toledo, Health Science Campus, HEB 274, 3000 Arlington Avenue, MS 1015, Toledo, OH 43614, USA

## Abstract

Primary cilia are nonmotile, microtubule-based, antenna-like organelles projecting from the apical surface of most mammalian cells. Elegant studies have established the importance of ciliary structure and function in signal transduction and the sensory roles of cilia in maintaining healthy cellular state. In particular, dysfunctional cilia have been implicated in a large number of diseases mainly characterized by the presence of fluid-filled cysts in various organs. Aside from polycystic kidney disease (PKD), however, the roles of cilia in polycystic liver disease (PLD), polycystic pancreas disease (PPD), and polycystic ovarian syndrome (PCOS) are still very vague. In addition, although gender and sex hormones are known to regulate cyst formation, their roles in regulating physiological functions of cilia need to be further explored.

## 1. Introduction

**Figure 1:**
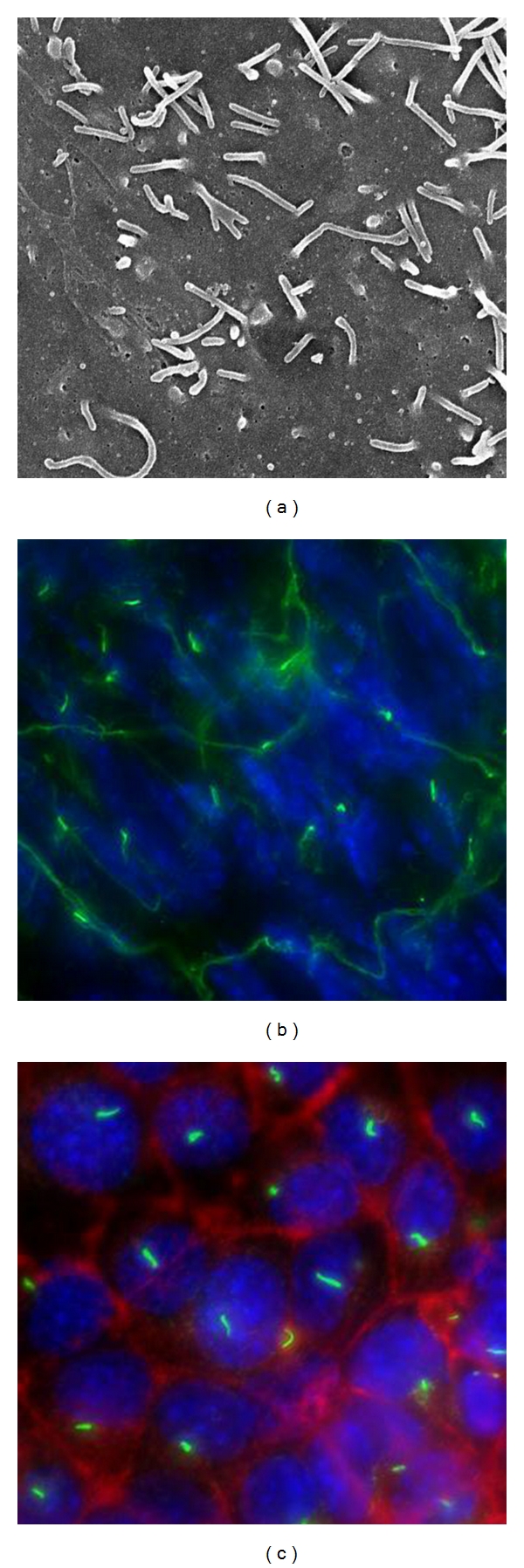
Primary cilia are present *in vivo* and *in vitro. *Primary cilia are present in all vestibular organs or tissues with vestibules (canals) that support perfusion of bodily fluid. Shown here are representative images demonstrating the presence of primary cilia in endothelial cells. (a) Scanning electron micrograph shows the presence of primary cilia in the lumen of mouse femoral artery. (b) Immunofluorescence image verify the presence of cilia in the mouse femoral endothelia. (c) When these endothelial cells were isolated, the cells retained their cilia in culture, as depicted in the image. Blue denotes cell nuclei; green represents acetylated-*α*-tubulin used as a cilia marker; red indicates actin cytoskeleton.

The primary cilium is an antenna-shaped organelle present on the apical surface of most mammalian cells (Figure 1). The main structural features of the primary cilium include a microtubule-based axoneme, which originates from the basal body or the mother centriole. Cilia play an important role in transmitting signals from the extracellular matrix to the cell interior, resulting in changes in gene expression and associated protein synthesis [1–3]. Their unique structures and locations help cells to detect and transmit even the minutest changes in the extracellular signals. Thus, cilia are important mechano- and chemosensory organelles [4, 5]. To assist in their sensory roles, cilia are bestowed with a large number of specialized proteins, known as “ciliary proteins,” which include receptors, ion channels, and secondary messengers; many of which localize to the ciliary body or the basal body [2]. Consequently, various studies in the past have shown that improper structure and/or localization of the ciliary proteins to the cilium and/or the basal body results in a special class of diseases, collectively termed as ciliopathies [6, 7].

**Figure 2:**
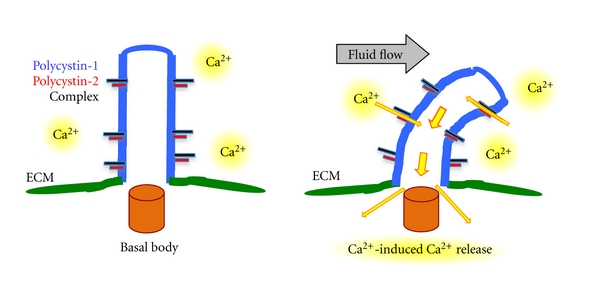
Mechanosensory cilia function involves calcium signal transduction. Mechanosensory cilia require functional polycystin complex. Fluid-flow-induced cilia bending will activate polycystin complex. This will mobilize calcium ions (Ca^2+^) influx from the extracellular matrix (ECM) into the cell. Calcium-induced calcium release will further activate various calcium-dependent proteins to maintain proper organogenesis.

Ciliary structure and function play an important role in mechanosensory function of the cilia [8–12]. The mechanosensory function of the cilium is involved in sensing fluid flow in many visceral organs such as kidneys, liver, pancreas, brain, spleen, bone, and others [2]. Primary cilia, expressed on the epithelial cells of these organs, sense fluid flow and transduce these signals into an intracellular calcium signaling response (Figure 2). Flow sensing is completely abolished in deciliated cells and in cells with dysfunctional polycystin-1 and polycystin-2. In the presence of fluid flow, cilia are activated resulting in a transient increase in intracellular calcium levels, which results in various cellular processes, including cell growth, differentiation, proliferation, and apoptosis [13–16]. Impaired mechanosensory function of cilium results in low levels of intracellular calcium, which then results in the activation of various cell proliferative pathways including cAMP, ERK, p-Akt (Ser^473^) pathways [17–20]. Abnormal regulation of these pathways promotes an increased cell proliferation resulting in cyst formation.

One of the most predominant ciliopathy arising due to impaired mechanosensory function of the primary cilium is polycystic kidney disease (PKD) [21–23]. Interestingly, PKD is also associated with cyst formation in other organs. Polycystic liver and pancreas associated with PKD have also been associated with abnormal cilia function or structure [24–27]. In this paper, we will briefly introduce PKD and focus on polycystic diseases in various organs, including the liver, pancreas, and ovary.

## 2. Polycystic Kidney Disease (PKD)

Autosomal dominant and autosomal recessive polycystic kidney disease (ADPKD and ARPKD) are two of the most common PKD diseases, which result in end-stage kidney failure in adults and children, respectively. ADPKD is caused by mutations in *PKD1 *(encoding polycystin-1) and *PKD2 *(encoding polycystin-2), while ARPKD arises due to mutations in *PKHD1 *(encoding fibrocystin) [28–30]. PKD is characterized by the presence of fluid-filled cysts in the kidneys, finally resulting in renal failure. Along with cystic manifestation, PKD patients and animal models also exhibit noncystic phenotype, including hypertension, left ventricular hypertrophy, abnormal arterial remodeling, intracranial aneurysm, among others. Not surprisingly, autopsy results of PKD patients show that more than 80% patients die of cardiovascular reasons than end-stage renal failure [31, 32].

**Figure 3:**
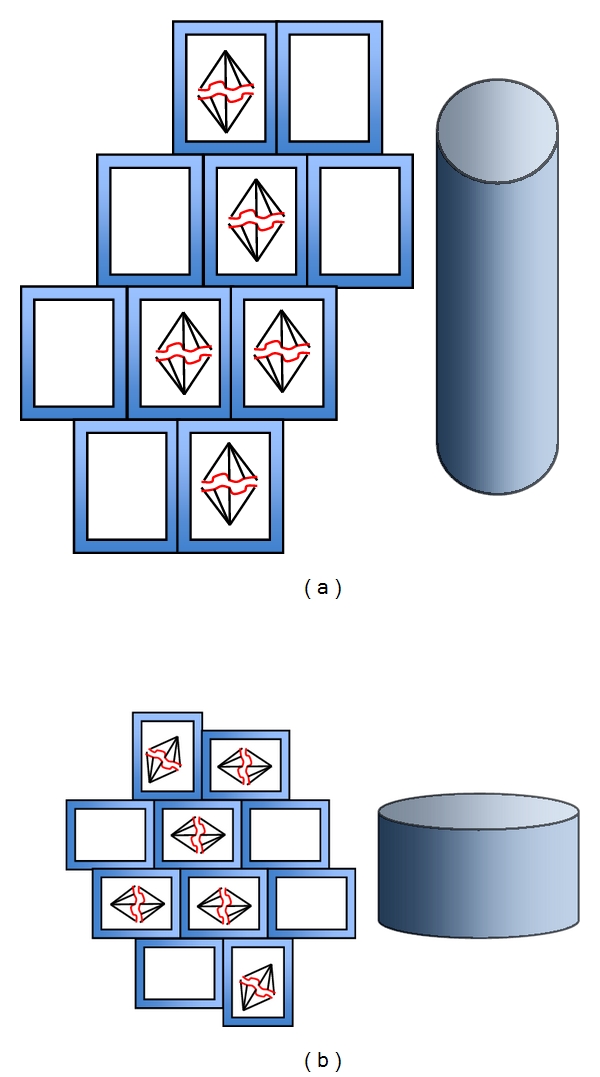
Defects in planar cell polarity results in cystogenesis. It is hypothesized that normal cilia function is required to control proper directional cell division. (a) Normal directional cell division is required to have an elongated tubular formation. (b) Disrupted directional cell division results in expanded formation of tubule.

Ciliary dysfunction tends to result in abnormal renal epithelial cells resulting in cyst formation and aberrant renal proliferation [33, 34]. It is further hypothesized that in healthy ciliated kidney cells, the mitotic spindle is oriented in an axis parallel to the longitudinal axis of the tubule [35, 36]. In PKD cells, however, a large number of cells exhibit randomized angle of the mitotic spindle, resulting in cyst formation (Figure 3).

### 2.1. Gender as a Factor

Incidence, prevalence, and progression of polycystic kidney diseases in humans and animal rodents are known to be dependent on gender [37–39]. Testosterone is renotropic in normal as well as diseased rodent models. Castrated male rodents exhibit limited disease progression in terms of renal size and cyst volumes. Interestingly, testosterone treatment of these castrated rats obviates the effect of castration. On the other hand, female rodents treated with testosterone exhibit increased cyst and kidney growth in both, females with and without ovariectomy [37]. Possible mechanisms for gender-related disparity observed could be due to differences in diet, renal mass or nephron number, systemic or glomerular hemodynamics and direct cellular effects of sex hormones. Men tend to have larger kidneys, in addition to more number of glomeruli than women [38]. Most importantly, sex hormones regulate various cytokines, growth factors, vasoactive agents, and extracellular matrix, such as nitric oxide, angiotensin, and collagen.

Detailed studies of the effect of androgens (male hormones) and estrogen (female hormones) indicate that androgens may be involved in stimulation of the renal angiotensin system (RAS) and endothelin system (ET-1) resulting in rapid cystic progression in males. Androgens also cause a downregulation of VEGF system and nitric oxide bioavailablity, resulting in increased cardiovascular problems in male PKD rodent models and male patients. On the other hand, estrogen seems to play a protective role in females. Estrogen has been found to suppress both RAS and ET-1 systems and upregulate VEGF system resulting in decreased loss of renal structure and associated renal function, along with reduced severity of cardiovascular effects [39].

A more recent study on the effect on mammalian target of rapamycin (mTOR) pathway indicates the role of gender and sex hormones in the treatment of PKD [17]. Male Han : SPRD rats treated with rapamycin exhibited a decreased proliferation of cystic tubules along with inhibition of renal enlargement, cystogenesis, and kidney failure by activation of the mTORC1 pathway. Female Han : SPRD rats of the same age treated with rapamycin for the same length of time as the male rats did not show any improvement in cystogenesis or kidney failure like male rats. Rapamycin was found to inhibit the proliferative p-Akt (Ser^473^) activity. In females, though mTORC1 pathway was activated in presence of rapamycin similar to males, rapamycin increased the proliferative p-Akt (Ser^473^) activity. This differential effect in female rodents could be explained on the basis of the female sex hormones, which are known to play a protective role in disease progression in female PKD patients as well as female rodents. However, further studies on castrated male animal models and ovariectomized female animal models are required to confirm the role of androgens and estrogens on rapamycin treatment of PKD patients.

### 2.2. Fertility Issues

Because PKD patients exhibit cysts in the male and female reproductive organs, fertility could become an issue. Several abnormalities have been observed in both men and women suffering from PKD. Infertility in male PKD patients mainly arises due to necrospermia or low sperm mortality and cysts in the seminal vesicles and ejaculatory ducts [40–42]. In addition, sperm motility is an issue in many PKD male patients. Sperms normally express 9 + 2 (motile) cilia, required for motility. However, large numbers of sperms in PKD patients express the 9 + 0 primary (immotile) cilia, which lack the central microtubule rods essential for motility, with some patients exhibiting only immotile cilia and hence immotile sperms [43]. Patients expressing completely immotile sperms were unable to father children with *in vitro* fertilization or intracytoplasmic sperm injections [44]. On the other hand, women suffering from PKD have not shown any specific fertility problems. This could be due to the fact that hypertension, compromised renal functions generally start after normal reproductive age in female PKD patients [45, 46].

### 2.3. Hormone Replacement Therapy

Irrespective of age, ovarian cyst is not found to be a major concern in PKD female patients [47, 48]. However, the use of hormone replacement therapy in postmenopausal PKD patients resulted in liver enlargements in most of these patients. Given that the most common extrarenal manifestation of PKD is hepatic cysts [49–51], it is not surprising that hepatic cysts occur more often, with more severity and at a younger age in female than male PKD patients. Nearly 80% female PKD patients exhibit hepatic cysts even with improved management of the diseases. These patients exhibit complications such as cyst infection, bleeding, or neoplasia [48, 51]. Endogenous and exogenous estrogen has been implicated in the severity of liver cysts in female PKD patients. In particular, pregnant PKD patients are at risk of developing massive hepatic cysts.

## 3. Polycystic Liver Disease (PLD)

Though cystic liver is one of the most common extrarenal manifestations observed in PKD, it also exists as an isolated inherited cystic disease, without any kidney cysts. PLD is characterized by the presence of cysts in the liver caused by proliferation and fluid secretion in cystic epithelial along with remodeling of the extracellular matrix around the cysts. PLD arises due to mutations in *PPRKCSH* or *SEC63 *[52, 53]. *PPRKCSH* encodes the noncatalytic *β*-subunit of glucosidase II (GII*β*) involved in the folding of glycoproteins, whereas *SEC63* encodes a protein product, which helps nascent peptides to translocate across the endoplasmic reticulum to become secreted- or membrane-bound proteins [54–56]. Though both *PPRKCSH* and *SEC63* protein products are not known, as yet, to colocalize to the primary cilia and/or the basal body, mutations in these two genes cause aberrant maturation of newly synthesized glyocoproteins, including polycystins. Overexpression or deletion of *PPRKCSH* in zebrafish results in developmental changes similar to those induced by imbalanced polycystin-2 [57, 58]. Rodent model studies with aberrant *Prkcsh*, *Sec63*, *Pkd1*, *Pkd2, *and *Pkhd1* genes indicate that cyst formation can generally be modulated by altering the expression of *Pkd1*, implying that polycytin-1 plays a central or rate-limiting role in both PLD and PKD. This further implies that PKD and PLD could share a common pathogenic pathway, even though PKD manifests in both liver and kidneys while PLD manifests only in the liver.

## 4. Polycystic Pancreas Disease (PPD)

The pancreas, involved in secretion of hormones and gastric enzymes, contains a maze of tubules and ducts involved in carrying the enzymes to the intestinal lumen. Ductal epithelial cells secrete bicarbonate to neutralize the acidic chime from the stomach. Pancreatic epithelial cells express primary cilia involved in mechanosensing of luminal flow and help maintain appropriate luminal dimensions [24, 25, 59, 60]. In addition, mice models with mutated hepatocyte nuclear factor-6 (HNF-6) indicate a significant decrease in the expression levels of ciliary proteins (i.e., cystin and fibrocystin) in the pancreas [61]. Thus, it has been proposed that HNF-6 is essential for activation of genes that activate epithelial cell polarity and formation of primary cilia in pancreatic cells.

## 5. Polycystic Ovarian Syndrome (PCOS)

PCOS can be classified as an endocrinal heterogeneous disorder affecting about 20% of women in the reproductive age. Hyperandrogenism, detected in 70% of PCOS patients, has been projected as one of the most important causes of PCOS [62–64]. This further results in long-term reproductive consequences, including lack of ovulation, suppression of gonadotropins, and development of cystic follicles in adulthood. Several studies have indicated an association between multiple genetic and environmental factors to be responsible for PCOS [65–67]. PCOS is associated with polycystic ovaries and chronic oligoanovulation, along with depression, mood disorders, obesity, hirsutism, and insulin resistance. In addition, women with PCOS are predisposed to high levels of high-density lipoprotein cholesterol, total cholesterol, low levels of low-density lipoprotein cholesterol along with cardiovascular disorders, and type-2 diabetes [68–71].

At present, no specific gene has been implicated in the pathogenesis of PCOS, though a wide category of genes are being studied based on the phenotypes observed, including genes correlated to androgen biosynthesis/actions, insulin resistance, inflammatory cytokines, and others [72–76]. Recent studies have identified that *DKK1* and *DNAJB1* are differentially expressed in PCOS tissue [76]. Thus, *DKK1 *(encoding a dickkopf related protein) and *DNAJB1* (encoding DnaJ or Hsp40 homolog) are potential genes of interest in the pathogenesis of PCOS.

*DKK1*, which is overexpressed in cultured ovarian theca from PCOS patients, has been shown to play important roles in embryogenesis and cell cycle regulation [76, 77]. On the other hand, *DNAJB1* is underexpressed in ovaries of PCOS patients and has important roles in protein folding, protein assembly-disassembly, and protein transport across cell membranes, especially in androgen signaling pathways. Of these two genes, *DNAJB1* has been identified and replicated as a gene of interest with respect to insulin resistance in PCOS. Thus, *DNAJB1* could affect androgenic pathways in PCOS.

Management of PCOS depends on the symptoms and mainly includes diet, weight management, exercise, and bariatric surgery in morbidly obese patients. In addition, low anovulation in PCOS patients results from low follicle stimulating hormone resulting from excess levels of luteinizing hormone, insulin, and/or androgen. This is generally treated with a variety of medications including estrogen receptor antagonists, tamoxifen, aromatase inhibitors, glucocorticoids, or gonadotropins. Androgen-related problems such as hirsutism, acne, and/or alopecia are generally treated with antiandrogens that either bind androgen receptors or decrease androgen production. Alternative medicines including kinesiology, herbalism, homeopathy, reflexology, acupressure, acupuncture, and massage therapy seem to be effective treatment in PCOS [78, 79].

In a few cases, PCOS has been reported in female PKD patients [80]. However, it is suggested that PKD patients and their unaffected relatives do not exhibit elevated risks of PCOS. This study includes a broad spectrum of female PKD patients, including premenopausal and postmenopausal female PKD patients. More specifically, ultrasound scanning of these women shows no difference of ovarian volume or frequency of ovarian cysts compared to control non-PKD group. In addition, fertile-age women with PKD do not exhibit impaired fertility [47, 48, 81].

## 6. Summary

Ciliopathy has been associated with cyst formation in various organs, including kidney, liver, and pancreas. However, the cellular and molecular roles of cilia are still vague. There is no doubt that future studies are critically needed to look at cystogenesis more extensively in various organs. In addition, further understanding on the physiological roles of cilia is undoubtedly necessary. For example, it is still a mystery how gender and sex hormones play a key role in the prevalence and progression of these polycystic diseases. If primary cilia play an important role in cystogenesis, we have no choice but to investigate how cilia function or structure is altered by hormonal regulation in our body.
